# Breast Cancer Antiestrogen Resistance 3 (BCAR3) Promotes Cell Motility by Regulating Actin Cytoskeletal and Adhesion Remodeling in Invasive Breast Cancer Cells

**DOI:** 10.1371/journal.pone.0065678

**Published:** 2013-06-06

**Authors:** Ashley L. Wilson, Randy S. Schrecengost, Michael S. Guerrero, Keena S. Thomas, Amy H. Bouton

**Affiliations:** 1 Department of Microbiology, Immunology & Cancer Biology, University of Virginia, Charlottesville, Virginia, United States of America; 2 Department of Cancer Biology, Thomas Jefferson University, Philadelphia, Pennsylvania, United States of America; China Medical University, Taiwan

## Abstract

Metastatic breast cancer is incurable. In order to improve patient survival, it is critical to develop a better understanding of the molecular mechanisms that regulate metastasis and the underlying process of cell motility. Here, we focus on the role of the adaptor molecule Breast Cancer Antiestrogen Resistance 3 (BCAR3) in cellular processes that contribute to cell motility, including protrusion, adhesion remodeling, and contractility. Previous work from our group showed that elevated BCAR3 protein levels enhance cell migration, while depletion of BCAR3 reduces the migratory and invasive capacities of breast cancer cells. In the current study, we show that BCAR3 is necessary for membrane protrusiveness, Rac1 activity, and adhesion disassembly in invasive breast cancer cells. We further demonstrate that, in the absence of BCAR3, RhoA-dependent signaling pathways appear to predominate, as evidenced by an increase in RhoA activity, ROCK-mediated phosphorylation of myosin light chain II, and large ROCK/mDia1-dependent focal adhesions. Taken together, these data establish that BCAR3 functions as a positive regulator of cytoskeletal remodeling and adhesion turnover in invasive breast cancer cells through its ability to influence the balance between Rac1 and RhoA signaling. Considering that BCAR3 protein levels are elevated in advanced breast cancer cell lines and enhance breast cancer cell motility, we propose that BCAR3 functions in the transition to advanced disease by triggering intracellular signaling events that are essential to the metastatic process.

## Introduction

Metastatic breast cancer is currently incurable and associated with a 5-year survival rate of only 23% (American Cancer Society). Thus, understanding the molecular mechanisms underlying metastasis is critical for improving patient survival. Cell motility is inherent to metastasis, and involves a complex, yet tightly regulated, series of events that promote remodeling of cellular adhesions and the actin cytoskeleton. Cells move directionally by first establishing protrusions toward a given stimulus. The actin-rich protrusions at the leading edge are then stabilized by nascent adhesions that are reinforced by tension generated from the actin cross-linking activity of myosin II. This rise in intracellular tension promotes adhesion disassembly in the rear and provides the force required to move cells along substrates within their microenvironment [1], [2], [3].

The Rho-family of GTPases, including Rac1 and RhoA, regulate actin cytoskeletal and adhesion dynamics as well as contractility. During cell migration, Rac1 promotes actin polymerization, membrane protrusions, and the formation of nascent adhesions, while RhoA creates intracellular tension by promoting actin bundling (stress fibers) and adhesion maturation [4]. RhoA has two major downstream effectors: the serine/threonine RhoA-associated kinase ROCK phosphorylates the regulatory light chain of myosin II (MLC II) to promote intracellular tension and acto-myosin contractility, while mammalian *Diaphanous* 1 (or mDia1) assembles and stabilizes actin to support adhesion maturation [4], [5], [6]. Although Rac1 and RhoA often appear to have opposing functions [7], their coordinate signaling is essential for cell motility [8].

The guanine nucleotide exchange factors (GEFs) and GTPase activating proteins (GAPs) that regulate Rho GTPases are often recruited to adhesions by specific adaptor/scaffolding molecules and kinases [3], [9]. In this work, we focus on the adaptor molecule Breast Cancer Antiestrogen Resistance 3 (BCAR3), which has emerged as an important regulator of breast cancer cell migration and invasion [10]. BCAR3, a member of the novel SH2 domain-containing protein (NSP) family, is overexpressed in breast cancer cell lines representative of more advanced, invasive breast cancers [10], [11]. BCAR3 is a binding partner of the adaptor molecule p130^Cas^ (Cas), which is a potent activator of Rac1 through its ability to couple with the adaptor molecule CrkII (Crk) and its associated GEF, DOCK180/ELMO [12], [13], [14]. BCAR3 has also been shown to promote interactions between Cas and the protein tyrosine kinase c-Src, leading to increased c-Src kinase activity and Cas phosphorylation. This, in turn, has significant implications in cell survival, proliferation and motility [15], [16], [17], [18].

In this study, we set out to determine the mechanism through which BCAR3 promotes breast cancer cell motility by examining its function in the regulation of membrane protrusions, adhesion turnover, and contractility. We show that BCAR3 is a positive regulator of Rac1 activity, membrane protrusiveness, and adhesion turnover in invasive breast cancer cells. When BCAR3 is selectively depleted, RhoA activity is increased and cells exhibit a highly contractile phenotype marked by prominent stress fibers, an increase in ROCK-mediated MLC II phosphorylation, and large ROCK/mDia1-dependent focal adhesions. Based on these data, we suggest that BCAR3 controls the balance between Rac1 and RhoA signaling in invasive breast cancer cells and, through this activity, functions as a positive regulator of actin cytoskeletal/adhesion remodeling and cell motility. Considering that BCAR3 is elevated in advanced breast cancer cell lines and enhances cell motility, we propose that BCAR3 upregulation may be a critical regulator of metastatic progression.

## Results

### BCAR3 promotes membrane protrusiveness

**Figure 1:**
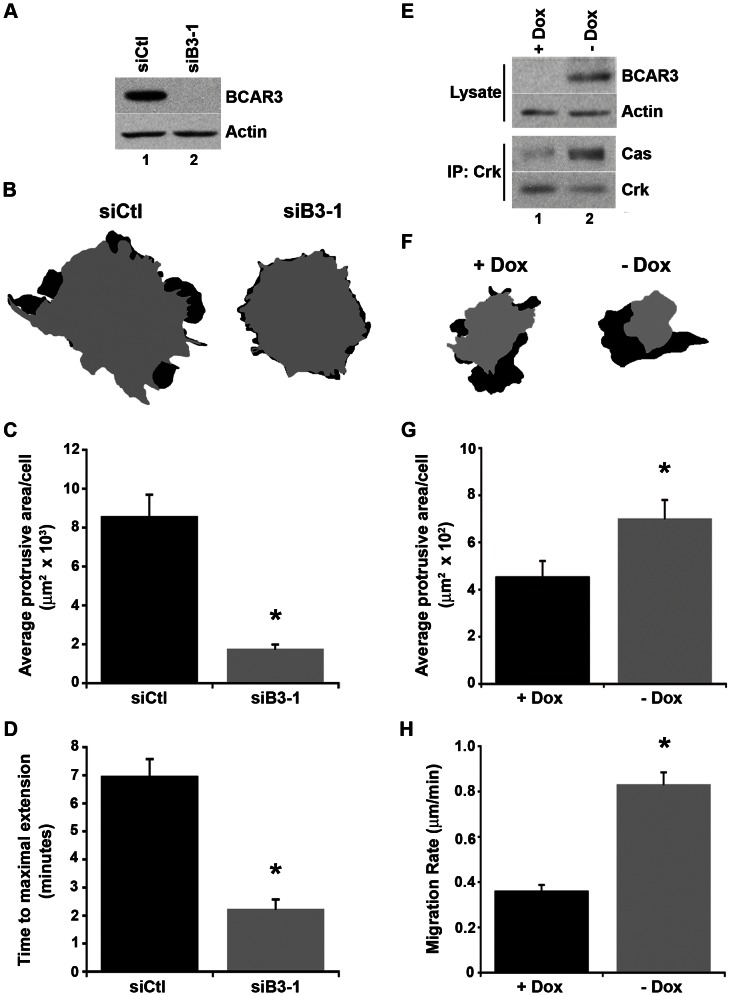
BCAR3 promotes membrane protrusiveness in breast cancer cells. (**A**) BT549 cells were transfected with control (siCtl; lane 1) or BCAR3-specific (siB3-1; lane 2) siRNA oligonucleotides and incubated for 72 hours prior to lysis. Representative immunoblots of total cell lysates are shown. (**B**) BT549 cells transfected as in (A) were plated on 10 µg/ml fibronectin for 4 hours and imaged by time-lapse phase microscopy for 12.5 minutes. Cell outlines of the first and last frames (pseudo-colored gray and black, respectively) of representative cells from Videos S1 and S2 are shown.(**C**) The average protrusive area was determined by measuring the area shown in black. Data represent the mean ± SEM of at least 12 cells over at least 4 videos (*, p<0.005). (**D**) The average time to maximal membrane extension was determined by kymography. Data represent the mean ± SEM of at least 12 kymographs over 3 separate videos (*, p<0.005). (**E**) MCF-7 cells expressing BCAR3 under the control of a tetracycline-inducible (Tet-off) promoter were treated in the presence (lane 1) or absence (lane 2) of 1 µg/ml doxycycline (Dox) for 72 hours. Total cell protein and Crk immune complexes were immunoblotted with the designated antibodies. (**F**) MCF-7 cells were treated with or without Dox as described in (E), then plated on 10 µg/ml fibronectin overnight and subjected to time-lapse DIC microscopy for 1 hour. Tracings generated as in (B) for representative cells in Videos S3 and S4 are shown. (**G**) The average protrusive area per cell was determined as in (C). Data represent the mean ± SEM of 31 cells per condition over 3 separate videos (*, p<0.02). (**H**) Cell motility was measured by tracing the movement of the nucleus over time (see Fig. S1). The average rate of migration was calculated by dividing the total distance traveled by time for each cell. Data represent the mean ± SEM of at least 72 cells per condition over 3 separate movies (*, p<0.0001).

Given that the establishment of membrane protrusions is a critical facet of cell migration [1] and the loss of BCAR3 has been shown to decrease breast cancer cell motility [10], we sought to determine the contribution of BCAR3 to membrane protrusiveness. BT549 cells, which are invasive breast cancer cells that express high levels of BCAR3, were transfected with control (siCtl) or BCAR3-specific (siB3-1) siRNA oligonucleotides and imaged by time-lapse video microscopy (**Videos S1 and S2**). BCAR3 protein levels were consistently reduced by greater than 90% in cells transfected with siB3-1 (**Fig. 1A**). To visualize the protrusive area of each cell, the first and last frames of the videos were pseudo-colored gray and black, respectively (**Fig. 1B**). Control cells developed one or more broad protrusions during the time span of the video, while BCAR3-depleted cells exhibited spiky, short-lived extensions. Both the average protrusive area per cell (**Fig. 1C**) and the time to maximal membrane extension (**Fig. 1D**) were significantly reduced in BCAR3-depleted cells.

Based on these results, we hypothesized that the converse should also be true, in that ectopic expression of BCAR3 in cells that normally express low levels of the protein would increase membrane protrusiveness and migration. To test this hypothesis, MCF-7 cells expressing BCAR3 under the control of a tetracycline-regulated promoter were imaged by time-lapse video microscopy (**Videos S3 and S4;** **Fig. 1E**, top panels; **Fig. 1F**). BCAR3 overexpression resulted in a significant increase in the average protrusive area per cell, a faster migration rate, and an increased distance traveled (**Figs. 1G and 1H** **;** **Fig. S1**).

Membrane protrusions are generated by dynamic actin remodeling through multiple pathways, including the Cas/Crk/Rac1 signaling axis [12], [14]. Previous studies by our group have shown that Cas tyrosine phosphorylation, which is required for Cas/Crk association, is increased upon BCAR3 overexpression [17]. Consistent with this finding, the amount of Cas present in association with Crk was found to be significantly elevated when BCAR3 was overexpressed (**Fig. 1E**, bottom panels). Thus, in addition to increasing membrane protrusiveness and migration, BCAR3 overexpression induces elevated Cas/Crk coupling.

### BCAR3 promotes membrane protrusiveness through activation of Rac1

**Figure 2:**
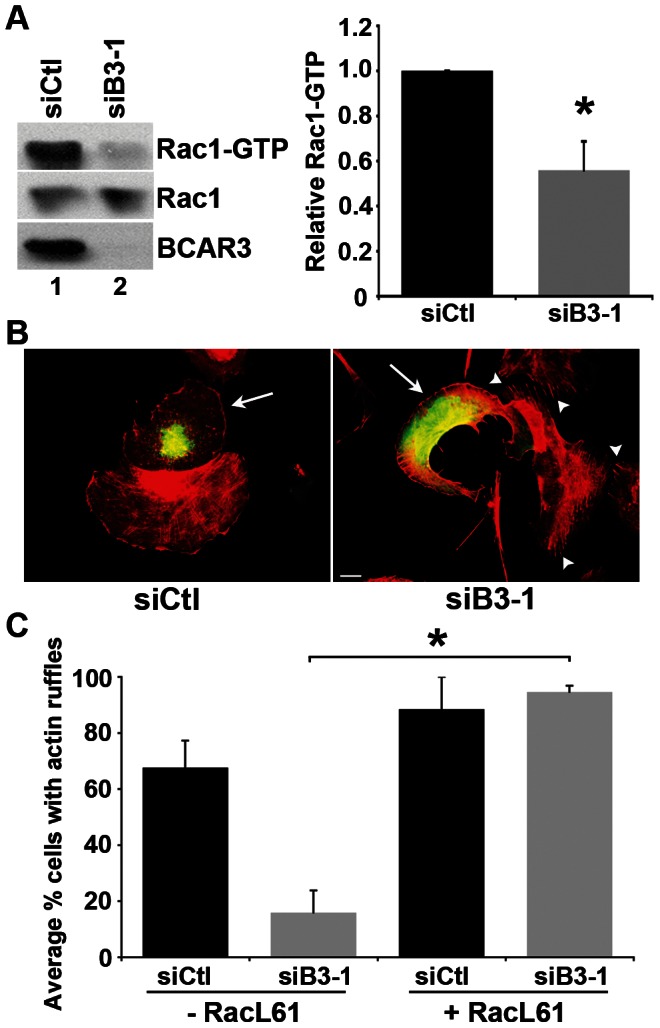
BCAR3 promotes Rac1 activity. (**A**) BT549 cells transfected with siCtl (lane 1) or siB3-1 (lane 2) siRNA oligonucleotides were incubated for 72 hours, held in suspension for 90 minutes, then plated on 10 µg/ml fibronectin for 1 hour. GTP-bound Rac1 was isolated from whole cell lysates by incubation with PAK-1-binding domain agarose. Bound proteins (top panel) and total Rac1 (middle panel) were detected by immunoblotting with a Rac1 antibody, and BCAR3 knockdown was confirmed with a BCAR3-specific antibody (bottom panel). Quantification of the relative GTP-Rac1 level is shown. Data represent the mean ± SEM of 3 independent experiments (*, p<0.05). (**B**) BT549 cells were transfected with siCtl or siB3-1 oligonucleotides, incubated for 24 hours, followed by transfection with plasmids encoding Myc-RacL61 for an additional 48 hours. Cells were plated onto 10 µg/ml fibronectin-coated coverslips for 1–3 hours and processed for immunofluorescence as described in the methods. Actin is stained with Texas red-conjugated phalloidin (red) and Myc (RacL61) with fluorescein isothiocyanate (FITC) (green). Arrows indicate Myc-RacL61 expressing cells. Arrowheads indicate actin-rich stress fibers. The images shown are representative of 6 separate experiments. Scale bar = 15 µm. (**C**) The percentage of cells exhibiting actin-rich ruffles was determined for non-transfected and RacL61-expressing cells. Data represent the mean ± SEM of at least 36 cells per condition over to 2 separate experiments (*, p<0.05).

Because Rac1 activity is required for membrane protrusions [19], we next investigated whether BCAR3 promotes membrane protrusiveness through its ability to modulate Rac1 activity [20]. To test this hypothesis, active GTP-bound Rac1 was measured in BT549 cells transfected with siCtl or siB3-1 oligonucleotides. While total Rac1 expression was equivalent in control and BCAR3-depleted cells, Rac1-GTP levels were significantly decreased in the absence of BCAR3 (**Fig. 2A**). To determine whether this decrease in Rac1 activity was responsible for the loss of protrusiveness seen in the absence of BCAR3, constitutively active Rac1 (Myc-RacL61) was transiently expressed in control and BCAR3-depleted cells and actin-rich membrane protrusions were visualized by immunofluorescence microscopy. As expected, BCAR3 depletion reduced the percentage of cells exhibiting protrusions in the absence of RacL61 (**Fig. 2C**). However, while expression of RacL61 in control cells did not have a significant effect on membrane protrusions (**Fig. 2B**, left panel, compare cell marked with arrow to adjacent cell), RacL61 expression in BCAR3-depleted cells significantly increased the percentage of cells containing membrane protrusions (**Fig. 2B**, right panel, compare cell marked with arrow to adjacent cells marked with arrowheads; **Fig. 2C**). Interestingly, BCAR3-depleted cells that did not express RacL61 (**Fig. 2B**, right panel, arrowheads) exhibited prominent actin-rich stress fibers that were not evident in control cells or BCAR3-depleted cells expressing constitutively active Rac1. Our group has reported this stabilization of stress fibers in the absence of BCAR3 previously [10]. Collectively, these data show that BCAR3 promotes membrane protrusions through a Rac1-dependent mechanism.

### BCAR3 alters actin cytoskeletal and adhesion remodeling

**Figure 3:**
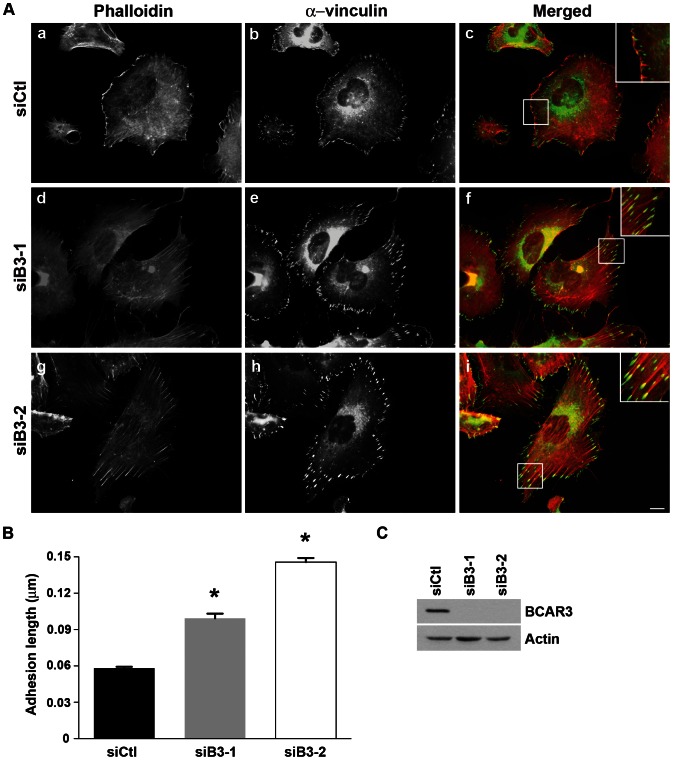
BCAR3 alters actin organization and adhesion size and distribution in invasive breast cancer cells. (**A**) BT549 cells were transfected with siCtl, siB3-1, or a smartpool consisting of 4 BCAR3-specific siRNA (siB3-2) oligonucleotides, incubated 72 hours, re-plated onto 10 µg/ml fibronectin-coated glass coverslips for 3 hours, and then processed for immunofluorescence as described in the methods. Actin and vinculin-containing adhesions were visualized with phalloidin (red) and vinculin (green) antibodies, respectively. Merged images are shown in the right panels, with insets showing higher magnifications of cell peripheries. Scale bar = 15 µm. A similar adhesion phenotype was observed with paxillin (unpublished data). (**B**) Vinculin-containing adhesions in siCtl (black bar), siB3-1 (gray bar), and siB3-2 (white bar) treated cells were measured in ImageJ. Data represent the mean ± SEM of at least 136 adhesions from at least 6 cells for each condition (*, p<0.0001). Asterisks indicate values that are significantly different from the control condition. (**C**) BT549 cells were transfected as described in (A). Representative immunoblot is shown confirming knockdown of BCAR3 using 2 distinct siRNA oligonucleotides.

**Figure 4:**
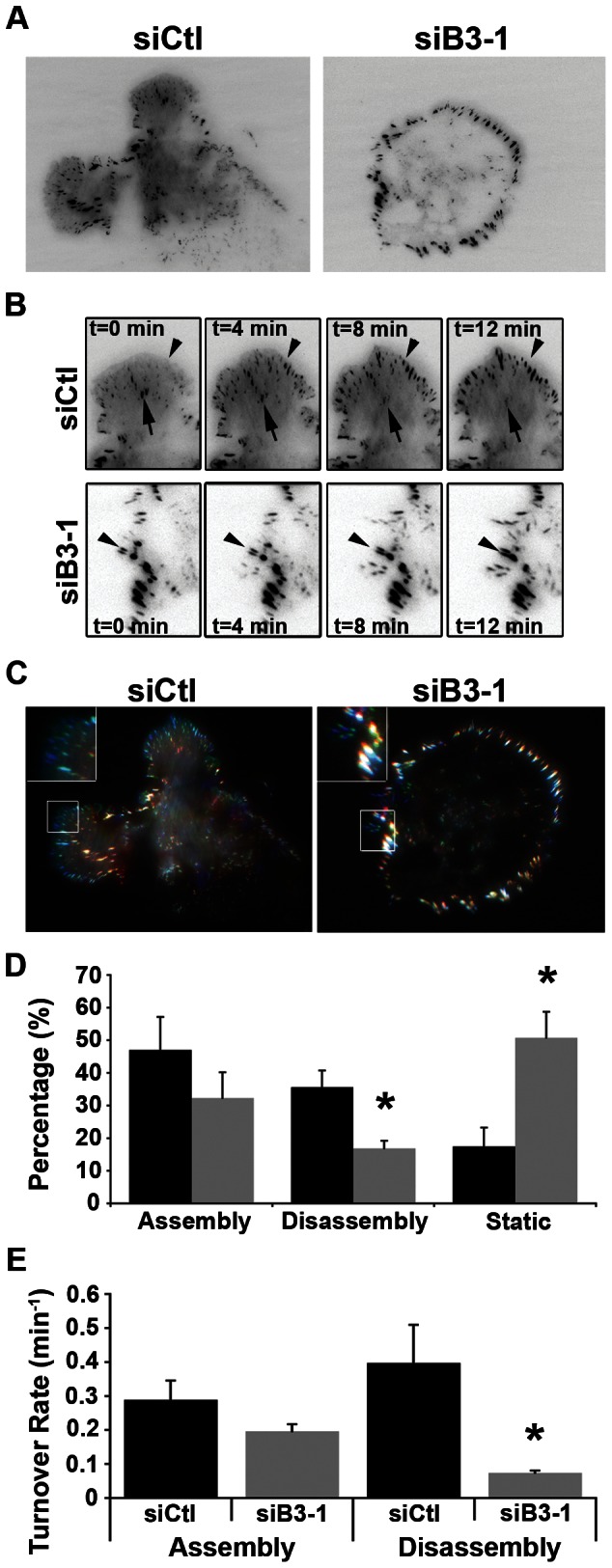
BCAR3 regulates adhesion disassembly. (**A**) BT549 cells were transfected with siCtl or siB3-1 siRNA oligonucleotides, incubated for 24 hours, and then transfected with plasmids encoding GFP-vinculin for an additional 48 hours. Cells were plated on 2 µg/ml fibronectin for 4 hours and then visualized by TIRF-based video microscopy to analyze adhesion dynamics. Representative images from Videos S5 and S6 are shown. (**B**) Time-lapse images from TIRF microscopy show assembly (arrowheads) and disassembly (arrows) of vinculin-containing adhesions over the specified time course for control (top row) and BCAR3-depleted (bottom row) cells. (**C**) Analysis of vinculin-containing adhesion turnover. The first, middle, and final frames from TIRF-based Videos S5 and S6 were pseudo-colored red, green, and blue, respectively, and then merged into a single image to visualize adhesion dynamics. At least 3 cells per condition were pseudo-colored. Insets show higher magnifications of peripheral adhesions. (**D**) Quantitative analysis of the adhesions that assembled, disassembled, or remained static over the time course shown in (B) for control (black bars) and BCAR3-depleted (gray bars) cells (*, p<0.05). (**E**) Quantitative analysis of the turnover rate of vinculin-containing adhesions. At least 18 adhesions from 3 separate control (black bars) and BCAR3-depleted (gray bars) cells were measured as described in the methods (*, p<0.005). Asterisks indicate values that are significantly different from control conditions.

The presence of prominent stress fibers in BCAR3-depleted cells [10] suggests that BCAR3 may influence actin cytoskeletal remodeling. This was tested by transfecting BT549 cells with siCtl, siB3-1, or a BCAR3-specific siRNA smartpool (siB3-2) of oligonucleotides (**Fig. 3C**). The cells were allowed to spread on fibronectin for 3 hours and then actin and adhesion structures were visualized by immunofluorescence microscopy. In control cells, actin was present in peripheral ruffles and diffusely throughout the cytoplasm (**Fig. 3A**, panel a). Vinculin was localized adjacent to actin-rich foci in what appeared to be nascent focal complexes (panels b and c) [21]. When BCAR3 was depleted from these cells, prominent actin-rich stress fibers were present throughout the cytoplasm (panels d and g). The majority of these structures appeared to be dorsal stress fibers that originate from single vinculin-containing focal adhesions (panels e and f, h and i). As is the case for ventral stress fibers that have adhesions at both ends, dorsal stress fibers are highly contractile and contribute to intracellular tension [3]. Interestingly, the length of the adhesions in BCAR3-depleted cells was significantly increased compared to control cells (**Fig. 3B**), suggesting a defect in adhesion turnover. This was further investigated by total internal reflective fluorescence (TIRF)-based video microscopy using GFP-vinculin as a marker of adhesions (**Videos S5 and S6;** **Figs. 4A and 4B**). Images representing the first, middle, and last frames of the time-lapse TIRF videos were pseudo-colored red, green, and blue, respectively, and merged into a single color image to more readily visualize the dynamics of adhesion assembly and disassembly (**Fig. 4C**). In control cells, adhesion assembly (**Fig. 4B**, top row, arrowheads; **Fig. 4C**, green or blue adhesions) was most often observed at the periphery of the cell, while adhesion disassembly (**Fig. 4B**, top row, arrows; **Fig. 4C**, yellow or red adhesions) was observed in more centrally located regions of the cell. Adhesions in BCAR3-depleted cells were predominantly localized to the periphery of the cell and showed accumulation but little loss of GFP-vinculin over time (**Fig. 4B**, bottom row, arrowheads; **Fig. 4C**, white adhesions). To quantify adhesion assembly and disassembly rates, the pixel intensity of vinculin-containing structures was determined as a function of time. While the percentage of adhesions undergoing assembly was not statistically different between control and BCAR3-depleted cells, cells lacking BCAR3 contained a significantly reduced number of adhesions undergoing disassembly (**Fig. 4D**). This resulted in a greater number of adhesions remaining “static” or stable. Moreover, the few adhesions that were seen to undergo disassembly in cells lacking BCAR3 had a significantly slower turnover rate (**Fig. 4E**). Taken together, these data indicate that adhesion dynamics, particularly disassembly, are regulated by BCAR3 in invasive breast cancer cells.

### Growth factor-induced cytoskeletal remodeling is regulated by BCAR3

**Figure 5:**
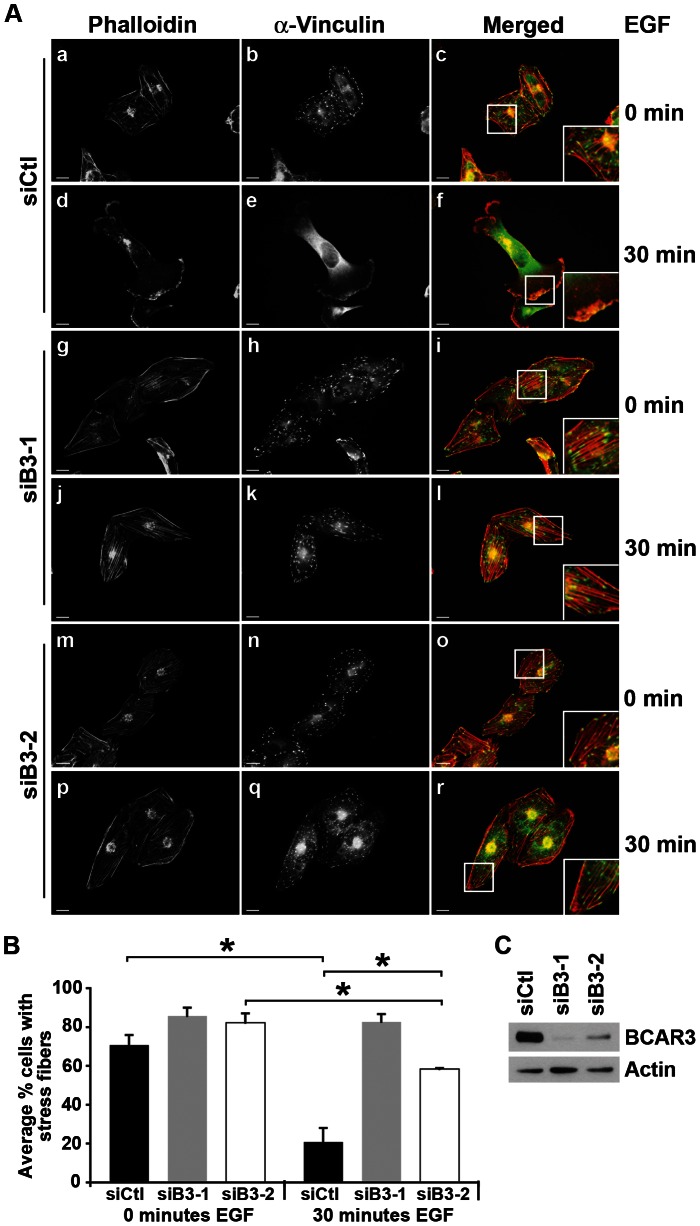
BCAR3 regulates actin cytoskeletal and adhesion remodeling in response to growth factor. (**A**) MDA-MB-231 cells were transfected with siCtl, siB3-1, or siB3-2 oligonucleotides and incubated for 24 hours prior to plating onto 10 µg/ml fibronectin-coated glass coverslips. Cells were serum-starved for 16–18 hours, stimulated with 100 ng/ml EGF for 0 or 30 minutes, and then fixed and processed for immunofluorescence as described in the methods. Actin and vinculin-containing adhesions were visualized with phalloidin (red) and vinculin (green) antibodies, respectively. Merged images are shown in the right panels; insets show higher magnifications of actin and adhesion structures. Scale bars = 15 µm. (**B**) The percentage of siCtl (black bars), siB3-1 (gray bars), and siB3-2 (white bars) treated cells containing actin-rich stress fibers was determined. Data represent the mean ± SEM of at least 730 cells per condition from 3 separate experiments (*, p<0.04). (**C**) MDA-MB-231 cells were transfected as described in (A). Representative immunoblots are shown confirming knockdown of BCAR3 using 2 separate siRNA oligonucleotides.

Thus far, we have shown that BCAR3 controls cytoskeletal changes that arise in response to cell adhesion to fibronectin. We previously reported that BT549 cells depleted for BCAR3 also failed to undergo characteristic cytoskeletal remodeling following growth factor stimulation (i.e. severing of actin-rich stress fibers and acquisition of membrane protrusions) [10]. Given these findings, we sought to define the extent to which BCAR3 regulated cytoskeletal dynamics in response to epidermal growth factor (EGF) stimulation. By using a second invasive breast cancer cell line (MDA-MB-231), we also sought to determine whether the impact of BCAR3 signaling on the actin cytoskeleton was consistent across multiple cell lines. MDA-MB-231 cells treated with control siRNA oligonucleotides exhibited robust actin cytoskeletal and adhesion remodeling in response to EGF, marked by a loss of stress fibers (**Fig. 5A**, compare panels a and d; **Fig. 5B**, black bars) and the redistribution of adhesions to sites of broad, actin-rich lamellipodia (**Fig. 5A**, compare panels b and c with panels e and f). In contrast, cells treated with siB3-1 siRNAs exhibited an attenuated response characterized by stabilization of stress fibers (**Fig. 5A**, panels g and j; **Fig. 5B**, gray bars) and large adhesions (**Fig. 5A**, panels h, i, k and l). Knockdown of BCAR3 in MDA-MB-231 cells using siB3-2 resulted in a similar, albeit somewhat less pronounced, defect in the cytoskeletal response to EGF (**Fig. 5A**, panels m–r; **Fig. 5B**, white bars). We attribute this difference to the fact that siB3-2 was less efficient at reducing BCAR3 expression in MDA-MB-231 cells than was siB3-1 (**Fig. 5C**). These data indicate that BCAR3 regulates actin cytoskeletal remodeling and adhesion dynamics in response to EGF as well as fibronectin.

### RhoA-mediated contractility predominates upon loss of BCAR3

**Figure 6:**
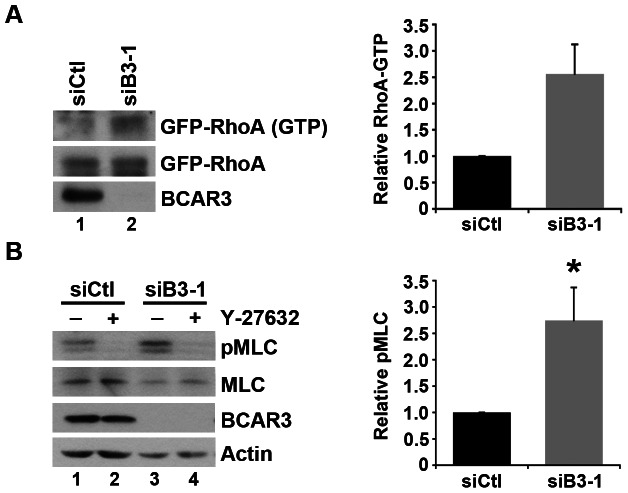
Loss of BCAR3 elevates RhoA activity and ROCK-mediated phosphorylation of MLC II. (**A**) BT549 cells were transfected with siCtl (lane 1) or siB3-1 (lane 2) siRNA oligonucleotides, incubated for 48 hours, followed by transfection with plasmids encoding GFP-tagged RhoA. Twenty-four hours later, cells were trypsinized, held in suspension for 90 minutes, and then plated on 10 µg/ml fibronectin for 1 hour. GTP-bound GFP-RhoA was isolated from whole cell lysates by incubation with Rhotekin binding domain (RBD) agarose. Bound proteins (top panel) and total GFP-RhoA (middle panel) were detected by immunoblotting with a Rho antibody, and BCAR3 knockdown was confirmed with a BCAR3-specific antibody (bottom panel). Quantification of the relative RhoA-GTP level is shown. RhoA activity was increased by an average of 2.6-fold±0.6 (n = 2). Error bars represent standard deviation. (**B**) BT549 cells transfected with siCtl or siB3-1 siRNA oligonucleotides were held in suspension for 90 minutes and then plated onto 10 µg/ml fibronectin in the absence or presence of 20 µM Y-27632. Cells were lysed in 2× boiling hot sample buffer, sheared with a 27-gauge needle, resolved by 12.5% SDS-PAGE, and immunoblotted with antibodies recognizing phospho-specific MLC (pThr18/pSer19) or total MLC (top panels). Total cell lysates were resolved by 8% SDS-PAGE and immunoblotted with antibodies recognizing BCAR3 and actin (bottom panels). Quantification of the relative pMLC level is shown. pMLC was increased by an average of 2.7-fold±0.6 (n = 5; *, p<0.05) in cells lacking BCAR3. Error bars represent SEM.

RhoA-dependent stress fibers and focal adhesions create intracellular tension, which is a hallmark of highly contractile cells [22]. Thus, our findings that BCAR3-depleted cells exhibit prominent stress fibers and mature adhesions in response to adhesion and growth factor signaling supports a hypothesis whereby RhoA activity is increased in the absence of BCAR3. To test this hypothesis, active GTP-bound RhoA levels were measured in control and BCAR3-depleted BT549 cells transiently expressing GFP-tagged RhoA. GTP-bound GFP-RhoA levels were increased by approximately 2.6-fold in the absence of BCAR3 (**Fig. 6A**). Downstream of RhoA, ROCK becomes activated and phosphorylates MLC II. Consistent with elevated RhoA/ROCK activity, phospho-MLC II (pMLC) levels were increased 2.7-fold over control cells when BCAR3 was depleted (**Fig. 6B**, compare lanes 1 and 3; see graph). As expected, MLC II phosphorylation was dependent on ROCK activity, since pMLC levels were nearly undetectable in the presence of the ROCK inhibitor Y-27632, irrespective of BCAR3 expression (lanes 2 and 4).

**Figure 7:**
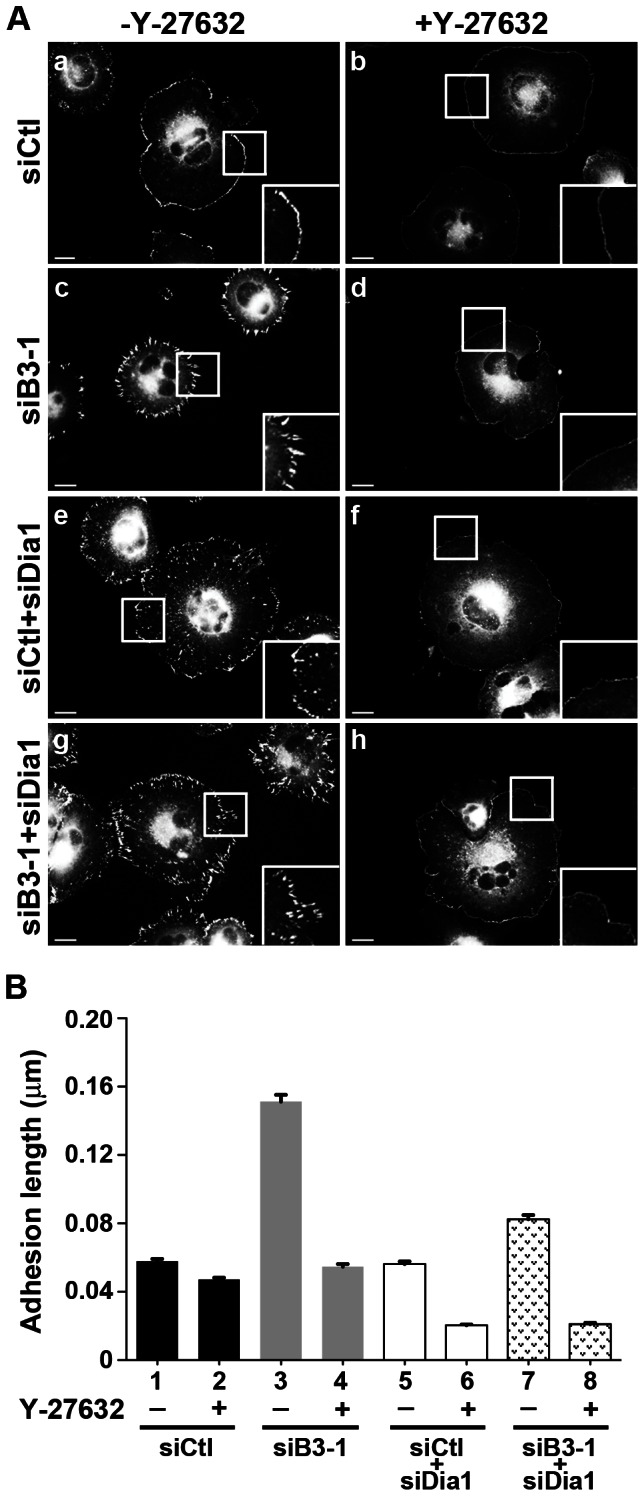
RhoA effector signaling mediates adhesion length in BCAR3-depleted invasive breast cancer cells. (**A**) BT549 cells transfected with siCtl or siB3-1 ± mDia1-targeted (siDia1) siRNA oligonucleotides were held in suspension for 90 minutes, plated onto 10 µg/ml fibronectin in the absence or presence of 20 µM Y-27632, and then processed for immunofluorescence as described in the methods. Adhesions were visualized using a vinculin antibody; insets show higher magnifications of peripheral adhesions. Scale bars = 15 µm. Similar results were obtained with paxillin staining (unpublished data). (**B**) Quantification of adhesion length in siCtl (black bars), siB3-1 (gray bars), siCtl+siDia1 (white bars), and siB3-1+siDia1 (hashed bars) treated cells in the absence or presence of Y-27632. Data represent the mean ± SEM of at least 343 adhesions from at least 13 cells from 2 separate experiments. ANOVA analysis confirmed all conditions were significantly different from one another (p<0.0001) except the following comparisons: siCtl-Y vs. siCtl+siDia1-Y (bars 1 vs. 5), siCtl+Y vs. siB3-1+Y (bars 2 vs. 4), and siCtl+siDia1+Y vs. siB3-1+siDia1+Y (bars 6 vs. 8).

ROCK signaling downstream of RhoA is also important for adhesion maturation. To investigate whether ROCK contributes functionally to the increased adhesion length present in cells depleted for BCAR3, vinculin-containing adhesions were examined under conditions in which ROCK activity was inhibited with Y-27632. As was shown in **Fig. 3**, adhesion length was significantly greater in cells depleted for BCAR3 (**Fig. 7A**, compare panels a and c; **Fig. 7B** compare bars 1 and 3). Inhibition of ROCK resulted in a reversal of this phenotype in BCAR3-depleted cells (**Fig. 7A**, panel d; **Fig. 7B**, compare bars 3 and 4), demonstrating that ROCK is required for the increase in adhesion length seen upon loss of BCAR3.

A second RhoA effector that has been implicated in adhesion maturation is mDia [6]. To determine whether mDia1 contributes to the adhesion response seen in BCAR3-depleted cells, BT549 cells were transfected with siCtl or siB3-1 along with mDia1-specific siRNAs (siDia1). In the presence of endogenous BCAR3, loss of mDia1 had no effect on adhesion size (**Fig. 7B**, compare bars 1 and 5), although the adhesions appeared more centrally located (**Fig. 7A**, compare panels a and e). In contrast, depletion of mDia1 in cells lacking BCAR3 diminished the elongated adhesion response seen in BCAR3-depleted cells, resulting in shorter adhesions (**Fig. 7A**, compare panels c and g; **Fig. 7B**, compare bars 3 and 7). This shows that, like ROCK, mDia1 contributes to the increased adhesion size observed under conditions of BCAR3 depletion. Interestingly, simultaneous inhibition/loss of ROCK and mDia1 resulted in adhesions that were markedly smaller than those present in control cells (**Fig. 7A**, compare panel a and f; **Fig. 7B**, compare bars 1 and 6). Moreover, this phenotype was maintained under conditions of BCAR3 loss (compare bars 6 and 8), demonstrating that dual blockade of the ROCK and mDia arms of RhoA signaling completely abrogates the effect of BCAR3 depletion on focal adhesion dynamics. Together, these data show that RhoA-dependent pathways predominate in invasive breast cancer cells in the absence of BCAR3.

## Discussion

**Figure 8:**
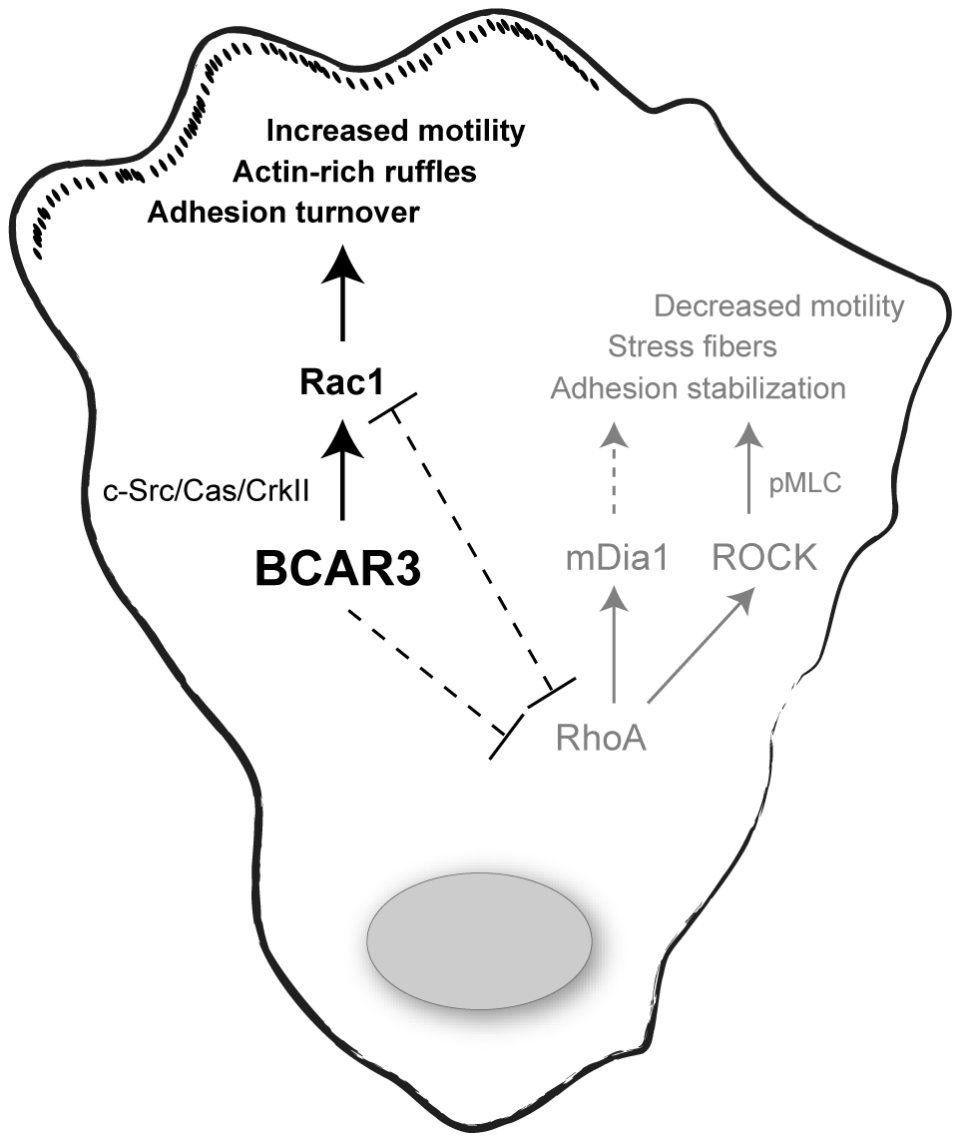
BCAR3 regulates the balance between Rac1 and RhoA signaling in invasive breast cancer cells. When BCAR3 is expressed at high levels, as is the case in invasive breast cancer cells, it promotes Rac1 activity, membrane protrusiveness, adhesion turnover, and rapid cell motility. When BCAR3 is depleted from these cells, RhoA activity is elevated and RhoA-dependent phenotypes predominate, resulting in the presence of prominent stress fibers, elevated pMLC levels, and large ROCK/mDia1-dependent focal adhesions. We suggest that BCAR3 may enhance Rac1 activity through c-Src/Cas/Crk coupling. It is also possible that BCAR3 may actively suppress RhoA activity/signaling, and in doing so, indirectly elevate Rac1 activity.

A balance between Rac1 and RhoA signaling is critical for cell motility. In cancer cells, the aberrant expression and/or activity of molecules that are responsible for regulating the activity of these GTPases can disrupt this balance and promote metastasis [23]. In this work, we show that BCAR3, an adaptor molecule that regulates cell motility and invasion, tips the balance in favor of Rac1 in invasive breast cancer cells, thus promoting Rac1-dependent events such as membrane protrusions and adhesion turnover (**Fig. 8**). The critical role played by BCAR3 in regulating this balance is underscored by the increase in RhoA activity and RhoA-dominant phenotypes (stable stress fibers, elevated pMLC, and large ROCK/mDia1-dependent focal adhesions) seen in these cells upon BCAR3 depletion.

### Regulation of Rac1 by BCAR3

BCAR3 shares structural and sequence homology with the Cdc25 family of Rac GEFs [24]. However, a recent report has shown that the C-terminal GEF-like domain adopts a “closed” conformation that is prohibitive for catalytic activity [25]. While it is therefore unlikely that BCAR3 functions as a GEF, it is possible that the Rac1 GEF DOCK180/ELMO becomes activated through BCAR3-dependent augmentation of Cas/Crk coupling [12], [13], [14], [26], [27] (**Fig. 8**). Our group has shown that elevated BCAR3 protein expression in breast cancer cells promotes c-Src/Cas interactions, c-Src kinase activity, c-Src-dependent Cas tyrosine phosphorylation, Cas/CrkII association, and Rac1 activity ([10], [17] and data herein). Additional data from Adam Lerner's group show that BCAR3/Cas interactions are required for c-Src to bind to, and phosphorylate, Cas as well as to promote optimal cell motility [18], [28]. Whether Cas is directly required for BCAR3-mediated Rac1 activation has been difficult to determine because we are unable to retain BCAR3 expression in the absence of Cas (data not shown). Our group is currently working to understand the mechanism underlying this regulation. However, it has been reported that direct binding between BCAR3 and Cas may be dispensable for Rac1 activation, as ectopic overexpression of a BCAR3 mutant that cannot bind to Cas is still able to promote elevated Rac1 activity in MCF-7 cells [28].

Recently, the receptor protein tyrosine phosphatase α (PTPα) has been shown to function as a molecular bridge that can serve as a link between adhesion signals and the BCAR3/Cas/c-Src signaling axis [29]. It is therefore interesting to note that PTPα-null cells share phenotypic and biochemical characteristics with BCAR3-depleted cells, including decreased adhesion-dependent Rac1 activity, c-Src/Cas interactions, c-Src kinase activity, Cas phosphorylation, Cas/Crk association, cell spreading, and migration/invasion [10], [17], [29]. Sun *et al.* suggest that PTPα/BCAR3 interactions are important for recruiting Cas to membrane-proximal regions of the cell [29], which could in turn augment Cas/c-Src interactions, c-Src activity, and ultimately Rac1 activation [17], [30]. This model emphasizes the importance of c-Src in mediating cytoskeletal responses to adhesion and growth factor signals, and helps to explain how BCAR3 may be an important regulator in these processes. It is interesting to speculate that the nature of the initiating adhesion signal (e.g. engagement of specific integrins and/or level of activation) may influence the temporal and spatial activation of this pathway.

Although not mutually exclusive, a second possibility that may account for the BCAR3-dependent Rac1 activity observed in invasive breast cancer cells is that BCAR3 may actively suppress RhoA signaling, leading indirectly to Rac1 activation (see **Fig. 8**). Indeed, there are numerous examples showing reciprocal regulation of Rac1 and RhoA signaling, such that when one GTPase is active, the other is suppressed [7]. This active suppression of RhoA by BCAR3 could arise from its ability to either positively regulate a Rho GAP and/or negatively regulate a Rho GEF. There are a number of candidate targets for this regulation. For example, the activity of p190RhoGAP is positively regulated by c-Src [31], making it a potentially attractive downstream target of BCAR3 signaling. There are additional candidate Rho GAPs (e.g. p250RhoGAP, DLC-1) and GEFs (e.g. p115RhoGEF, p190RhoGEF, LARG) downstream of integrins that are modulated by c-Src and other Src-family kinases [32], [33]. Future studies will determine whether any of these molecules contribute to the suppression of RhoA by BCAR3 and, in so doing, help to elucidate the mechanism by which BCAR3 affects the balance between Rac1 and RhoA signaling in invasive breast cancer cells. Finally, it is important to note that Rac1-RhoA reciprocity could not only account for Rac1 activation through suppression of RhoA, but the converse could also be true in that the high RhoA activity seen in BCAR3-depleted cells could result from diminished Rac1 activity (see **Fig. 8**).

### BCAR3 and breast cancer progression

BCAR3 protein expression is elevated in cell lines representative of triple-negative breast cancers compared to estrogen receptor-positive cells [10], [11]. As discussed above, BCAR3 function is intimately linked to two proteins, Cas and c-Src. Like BCAR3, these molecules are established regulators of cell motility, antiestrogen resistance, and other aggressive breast cancer behaviors [34], [35], [36], [37], [38], [39]. Interestingly, c-Src kinase activity is also elevated in triple negative breast cancers [40], [41]. Since BCAR3 has been shown to function through Cas to activate c-Src [15], [17], we suggest that its upregulation in triple negative breast cancer cells may contribute to the elevated c-Src activity seen in these tumors.

In addition to c-Src, EGFR is frequently overexpressed in aggressive breast tumors and triple negative breast cancer cell lines, as are a number of downstream components of EGF signaling pathways [42], [43], [44]. The data presented above showing that BCAR3 regulates the cytoskeletal response of invasive breast cancer cells to EGF thus provide a second point of convergence between BCAR3 and intracellular signaling pathways that control tumor cell motility and invasion. As is the case for the BCAR3/c-Src/Cas/Crk signaling axis, EGFR/BCAR3 signaling may contribute to actin remodeling through Rac1. However, a second potential mechanism involves the actin severing protein cofilin, which becomes activated in response to EGF and causes stress fiber dissolution to produce a pool of free actin monomers available for polymerization [42]. Thus, it will be important to explore the possibility that BCAR3 may also contribute to actin remodeling through this EGFR-cofilin pathway. There is considerable evidence for a role of EGF in promoting breast cancer cell invasion, and we have shown that BCAR3 can regulate the migration/invasion of breast tumor cells toward EGF [10], [45]. Whether this is achieved through the actin remodeling activities of BCAR3 is yet to be determined.

Despite evidence for BCAR3 as a regulator of invasive breast cancer cell motility and invasion, the role of BCAR3 in other cell types is not widely known. While BCAR3 mRNA is present in multiple cell types and tissues, its expression appears to be largely dispensable for development since BCAR3 knockout mice are born at the expected Mendelian frequency and have normal lifespans [46], [47]. In fact, the only spontaneous defect reported for these mice is in the lens of the eye [47]. Thus it is interesting that this molecule plays such an essential role in regulating cytoskeletal remodeling, adhesion turnover, and cell motility in invasive breast cancer cells. We hypothesize that BCAR3 expression may become upregulated in breast cancer cells in response to selective pressures present in the tumor microenvironment such as hypoxia or nutrient deprivation. The BCAR3 signaling pathway would then be in place to promote rapid and efficient invasion/migration of these tumor cells to distal sites in response to these environmental stresses. Importantly, our finding that these cells fail to respond properly to chemical (e.g. EGF) and physical (e.g. adhesion signals) stimuli in the absence of BCAR3 could have significant implications for treatment of breast cancers that express this protein, as it may be possible to target BCAR3 (or other molecules within the BCAR3/Cas/c-Src signaling network) in the tumors with limited collateral damage to other tissues. Future work is needed to determine the potential benefits of this type of an approach.

## Materials and Methods

### Antibodies, reagents and plasmids

The following monoclonal antibodies were used: β-Actin and vinculin (Sigma-Aldrich, St. Louis, MO); Rac1, mDia1 and Crk (BD Biosciences, San Jose, CA); Rho (Millipore, Billerica, MA); Myc (9E10) (Lymphocyte Culture Center, UVA). The following polyclonal antibodies were used: BCAR3 (Bethyl Laboratories, Inc., Montgomery, TX); pThr18/pSer19 MLC II and total MLC II (Cell Signaling Technology, Danvers, MA); FITC-conjugated goat anti-mouse and Texas red-conjugated goat anti-rabbit (Jackson ImmunoResearch Laboratories, Inc., West Grove, PA); CasB [48]. Texas red-conjugated phalloidin (Molecular Probes, Eugene, OR), EGF (Sigma), and ROCK inhibitor, Y-27632 (Calbiochem, Billerica, MA) were also used. Dr. A. R. Horwitz provided plasmids encoding Myc-RacL61, GFP-vinculin and GFP-RhoA (UVA).

### Cell Culture

BT549 and MDA-MB-231 cells (American Type Tissue Culture, Manassas, VA) and tetracycline-regulated MCF-7 cells stably expressing Myc-BCAR3 were cultured as previously described [10], [17].

### Small-interfering RNA and plasmid transfection

Cells were transfected with 20 µM of the following small-interfering RNA (siRNA) oligonucleotides using Lipofectamine RNAiMAX reagent (Invitrogen, Grand Island, NY) following the manufacturer's protocol: non-targeting control (siCtl; Ambion, Grand Island, NY), BCAR3 SH2-domain-targeting (siB3-1), BCAR3-targeting ON-TARGETplus SMARTpool (siB3-2), and mDia1-targeting ON-TARGETplus SMARTpool (siDia1) (Dharmacon. Lafayette, CO). The siB3-1 and siB3-2 oligonucleotides were described previously [10], [17]. Plasmid transfection of Myc-RacL61 or GFP-vinculin was performed using Fugene HD Transfection Reagent (Roche, Indianapolis, IN) following manufacturer's specifications. Plasmid transfection of GFP-tagged wildtype RhoA was performed using Lipofectamine 2000 (Invitrogen) following manufacturer's specifications.

### Immunoprecipitation, immunoblotting, and immunofluorescence

Cells were lysed in ice-cold radioimmune precipitation assay (RIPA) buffer and protein concentrations determined as previously described [10]. Immunoprecipitations and immunoblotting were performed as previously described [10]. Cells plated on fibronectin-coated glass coverslips were processed, visualized through a Nikon Eclipse TE2000-E microscope, and photographed as previously described [10].

### Live cell imaging

Cells were plated onto fibronectin-coated glass bottom dishes (BT549) or 35 mm Delta T dishes (Bioptechs, Inc., Butler, PA) (MCF-7) and cultured at 37°C, pH 7.4 in CCM1 media (Hyclone). For BT549 cells, phase images were captured every 5 seconds for 12.5 minutes on a light microscope (Diaphot, Nikon) with a video camera (KY-F55B, Victor Company of Japan). Images were then processed using MetaMorph Software (Molecular Devices, Sunnyvale, CA). For MCF-7 cells, phase images were captured every 30 seconds for 1 hour using an inverted microscope with a 20× differential interference contrast (DIC) objective, heated stage (Bioptechs, Inc.), and an ORCA camera. Images were then processed using Openlab software.

### Protrusion dynamics

To quantify protrusive behavior, total cell area at the first and final frame of a time-lapse movie was traced and pseudo-colored gray (first frame) or black (last frame). The average protrusive area was determined by measuring the area shown in black using ImageJ software (National Institutes of Health, Bethesda, MD). The average time (in minutes) to maximal membrane extension was determined by creating kymographs of cells from the time-lapse videos using ImageJ. The average distance traveled was determined in ImageJ by tracing nucleus movement of each cell over the course of the time-lapse sequence. The average rate of migration was calculated by dividing the total distance traveled by each cell by time.

### Adhesion dynamics

BT549 cells transfected with control or BCAR3-specific siRNAs and plasmids encoding GFP-vinculin were plated on fibronectin-coated glass bottom dishes and allowed to spread. Images were captured using an inverted TIRF microscope (1X70; Olympus) with a 60× objective and a cool charged-couple device camera (Retiga Exi; Qimaging). The fluorescence intensity of individual adhesions from cells expressing GFP-vinculin was measured over time as follows. Images were captured every 5 seconds using MetaMorph software. Adhesions located at the cell periphery and/or protruding edge were selected for analysis. ImageJ software was applied to the entire image stack to subtract the background fluorescence intensity and to correct for photobleaching. The assembly and disassembly of vinculin-containing adhesions were determined by measuring pixel intensity over time. Both the increase (assembly) and decrease (disassembly) in fluorescence intensity were linear as a function of time on semilogarithmic plots. The rate constants for the assembly and disassembly of vinculin-containing adhesions were determined from the slopes of these graphs. For each rate constant determination, measurements were obtained for 3–5 individual adhesions on 8–10 cells.

### GTP-bound GTPase pull-down assays

To measure GTP-Rac1, BT549 cells were transfected with siCtl or siB3-1 siRNA oligonucleotides, incubated for 72 hours, trypsinized, held in suspension for 90 minutes, and then plated on 10 µg/ml fibronectin for 1 hour. Cells were rinsed twice with ice-cold PBS and lysed in ice-cold RIPA buffer. GTP-bound Rac1 was isolated from whole cell lysates by incubation with PAK-1-binding domain agarose (Millipore) following manufacturer's instructions. To measure GTP-RhoA, BT549 cells were transfected with siCtl or siB3-1 siRNA oligonucleotides, incubated for 48 hours, and then transfected with plasmids encoding GFP-tagged RhoA. Twenty-four hours post-transfection, cells were trypsinized, held in suspension for 90 minutes, and then plated on 10 µg/ml fibronectin for 1 hour. Cells were then rinsed twice with ice-cold PBS and lysed in ice-cold magnesium lysis buffer and incubated with Rhotekin binding domain (RBD) agarose (Millipore) following manufacturer's instructions.

### Statistical Analysis

Two-tailed Student's *t* tests were used for the pair-wise comparison of two experimental groups. A Kruskal-Wallis (ANOVA) and Dunn's Multiple Comparison post-test were used to compare multiple experimental groups.

## Supporting Information

Figure S1 BCAR3 overexpression increases migration distance. (**A**) MCF-7 cells expressing endogenous (+Dox) or overexpressed levels (−Dox) of BCAR3 were imaged by time-lapse microscopy. Migration distance was determined by tracing the movement of the cell nuclei using ImageJ. Representative tracings from Videos S3 and S4 are shown. (**B**) Quantification of migration distance (*, p<0.05).
(TIF)

Video S1 BCAR3 regulates membrane protrusiveness. BT549 cells were transfected with a control siRNA oligonucleotide, plated on fibronectin for 4 hours, and then imaged by time-lapse phase microscopy using a light microscope (Diaphot, Nikon) with a video camera (KY-F55B). Frames were taken every 5 seconds for 12.5 minutes.
(MOV)

Video S2 BCAR3 regulates membrane protrusiveness. BT549 cells were transfected with a BCAR3-specific siRNA oligonucleotide, plated on fibronectin for 4 hours, and then imaged by time-lapse phase microscopy using a light microscope (Diaphot, Nikon) with a video camera (KY-F55B). Frames were taken every 5 seconds for 12.5 minutes.
(MOV)

Video S3 BCAR3 regulates protrusiveness and cell motility. MCF-7 cells expressing endogenous BCAR3 were plated on fibronectin overnight, followed by time-lapse microscopy using an inverted microscope (Nikon TE200) with a 20× DIC objective and heated stage (Bioptechs) with attached video camera. Frames were taken every 30 seconds for 1 hour.
(MOV)

Video S4 BCAR3 regulates protrusiveness and cell motility. MCF-7 cells overexpressing BCAR3 were plated on fibronectin overnight, followed by time-lapse microscopy using an inverted microscope (Nikon TE200) with a 20× DIC objective and heated stage (Bioptechs) with attached video camera. Frames were taken every 30 seconds for 1 hour.
(MOV)

Video S5 BCAR3 regulates adhesion dynamics. BT549 cells were transfected with a control siRNA oligonucleotide and plasmids encoding GFP-vinculin, plated on fibronectin for 4 hours, and then imaged by TIRF-based video microscopy to analyze adhesion turnover. Representative movie of GFP-vinculin containing adhesions visualized for 3 minutes.
(MOV)

Video S6 BCAR3 regulates adhesion dynamics. BT549 cells were transfected with a BCAR3-specific siRNA oligonucleotide and plasmids encoding GFP-vinculin, plated on fibronectin for 4 hours, and then imaged by TIRF-based video microscopy to analyze adhesion turnover. Representative movie of GFP-vinculin containing adhesions visualized for 3 minutes.
(MOV)
