# Supplementary figures and images for: Breast Cancer Antiestrogen Resistance 3 (BCAR3) Promotes Cell Motility by Regulating Actin Cytoskeletal and Adhesion Remodeling in Invasive Breast Cancer Cells
Source: PLoS One. 2013 Jun 6;8(6):e65678. doi: 10.1371/journal.pone.0065678 (PMC3675087; doi:10.1371/journal.pone.0065678)

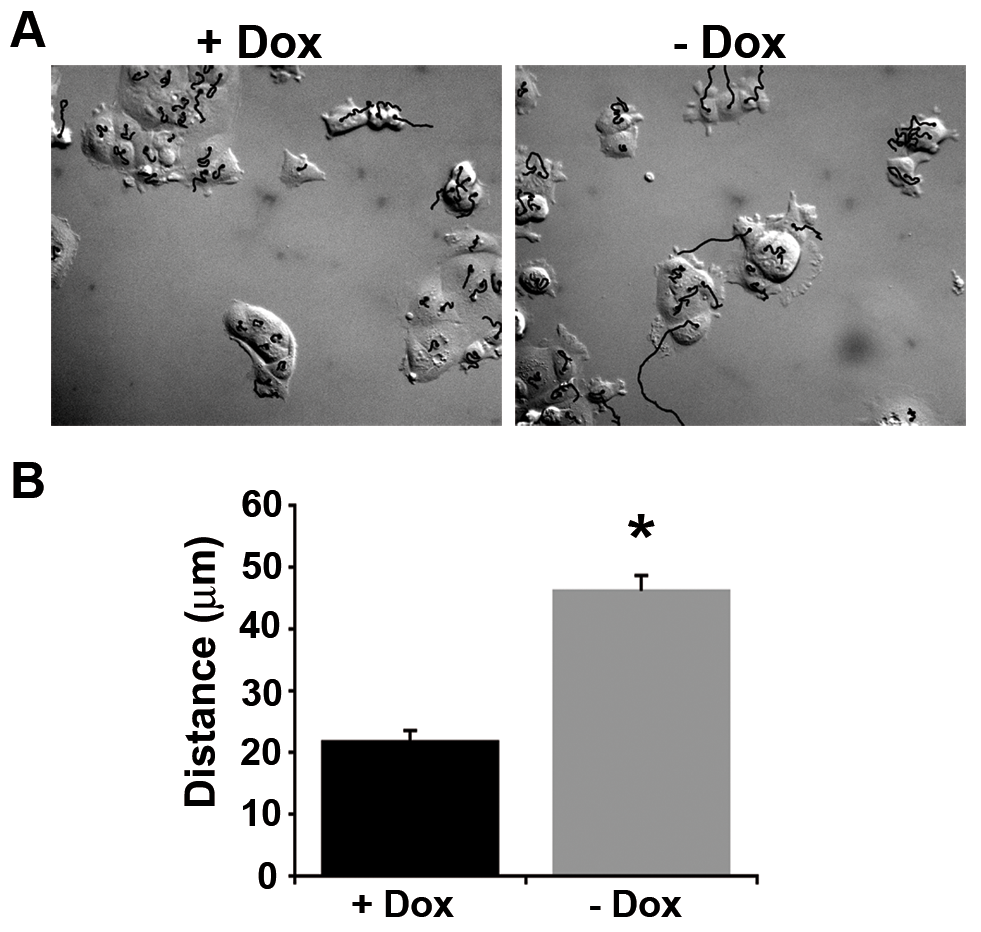

Supplement: Figure S1 — BCAR3 overexpression increases migration distance. (A) MCF-7 cells expressing endogenous (+Dox) or overexpressed levels (−Dox) of BCAR3 were imaged by time-lapse microscopy. Migration distance was determined by tracing the movement of the cell nuclei using ImageJ. Representative tracings from Videos S3 and S4 are shown. (B) Quantification of migration distance (*, p<0.05). (TIF) [file pone.0065678.s001.tif]
